# Ensuring That Marginalized Young People Feel Welcome, Understood, and Empowered in Health Services: A Qualitative Examination of the Service Needs of Aboriginal LGBTQA+ Young People

**DOI:** 10.1177/10497323251329765

**Published:** 2025-04-17

**Authors:** Shakara Liddelow-Hunt, Ashleigh Lin, Yael Perry, Braden Hill, Bep Uink

**Affiliations:** 1 School of Population and Global Health, 170517 University of Western Australia, Perth, WA, Australia; 2 117610 The Kids Research Institute Australia, Nedlands, WA, Australia; 3 Edith Cowan University, Mount Lawley, WA, Australia; 4 Kulbardi Aboriginal Centre, 5673 Murdoch University, Murdoch, WA, Australia; 5 Australian Indigenous Health*Info*Net, Kurongkurl Katitjin, Centre for Indigenous Australian Education and Research, 2498 Edith Cowan University, Joondalup, WA, Australia

**Keywords:** LGBTQA+;, Indigenous;, Aboriginal;, qualitative;, well-being;, mental health;, service provision

## Abstract

A lack of appropriate care and discrimination in healthcare settings likely compounds the existing risks to mental health and well-being for Aboriginal and Torres Strait Islander lesbian, gay, bisexual, trans, queer/questioning, and asexual (LGBTQA+) young people. The current study contributes findings from Aboriginal LGBTQA+ young people’s perspectives on their health service needs and preferences. Data consists of qualitative interviews and focus groups with *N* = 14 Aboriginal LGBTQA+ young people aged 14–25 years in Boorloo (Perth), Western Australia. The data was analyzed using reflexive thematic analysis. Analysis identified three major themes: (1) Unmet need for “whole self” care, (2) Communicating to young people that they will be welcome, safe, and cared for, and (3) Engaging communities to address structural inequalities. These findings shed light on the almost complete lack of Aboriginal LGBTQA+ youth-focused care available and point to the importance of health workers and, especially, mental health professionals understanding the broader sociohistorical context that impacts young people’s well-being. Ultimately, while many Aboriginal LGBTQA+ young people have positive experiences of receiving care for their health and well-being, there persists a feeling of being unable to wholly exist in healthcare settings.

## Introduction

Marginalization, enacted through an array of internalized, interpersonal, and structural stressors, is associated with poor mental health and well-being outcomes (Kairuz et al., 2021; Meyer, 2003; Paradies, 2006). However, service provision frequently mirrors existing inequalities and is not always appropriate or accessible to those who need it most, further contributing to disparity in health outcomes (Amos et al., 2023; Australian Institute of Health and Welfare, 2023; Australian Institute of Health and Welfare et al., 2006; Ben et al., 2017; Hill et al., 2023; Kelaher et al., 2014; Lyons et al., 2022; Temple et al., 2020). Existing at the intersection of two (or more) marginalized identities, Aboriginal and Torres Strait Islander lesbian, gay, bisexual, trans, queer/questioning, and asexual (LGBTQA+) people are not only likely subjected to double discrimination but it is also likely that existing interventions that target these priority populations separately may be ineffective for those at the intersection.

There has been very little empirical research concerning Aboriginal and Torres Strait Islander LGBTQA+ people’s well-being until recent years, despite numerous calls from community leaders for research in the area, highlighting in particular the risk for suicide among Aboriginal and Torres Strait Islander LGBTQA+ young people (Bonson, 2016; Burin, 2016; Clarke, 2017; Power, 2017; Somerville, 2018; Story Carter, 2017). Seperately, both Aboriginal and Torres Strait Islander people and LGBTQA+ people are at increased risk for a number of poor health outcomes, in particular poor mental health (Azzopardi et al., 2018; Meyer, 2003; Russell & Fish, 2016; Thurber et al., 2022). These poor outcomes are driven by marginalization (Meyer, 2003; Russell & Fish, 2016; Thurber et al., 2022), including a lack of appropriate healthcare and discriminatory experiences in healthcare settings leading to distrust and avoidance (Patton et al., 2016). The limited research specific to Aboriginal LGBTQA+ people points to experiences such as family violence (Aboriginal Health Council of South Australia, 2019; Dudgeon et al., 2015; Hill, Dodd, Uink, Bonson, & Bennett, 2022), physical assault (Hill et al., 2021), harassment (Hill, Dodd, Uink, Bonson, & Bennett, 2022), racial and cisheterosexist discrimination (Aboriginal Health Council of South Australia, 2019; Hill, Dodd, Uink, Bonson, & Bennett, 2022; Soldatic et al., 2021; Sullivan et al., 2021), and perceived pressure to suppress their sexuality or gender (Aboriginal Health Council of South Australia, 2019; Uink et al., 2020), all of which are associated with poor mental health in the broader literature.

These health inequities are further complicated by what Day et al. (2023, p. 3) identify as “…a help-seeking quandary for Aboriginal and Torres Strait Islander LGBTQIASB+ people who face barriers to seeking care at both Aboriginal and Torres Strait Islander and LGBTQIA+ specific services.” Aboriginal and Torres Strait Islander LGBTQA+ people may be reluctant to engage with services because of previous experiences of discrimination (Day et al., 2023) or, conversely, be referred away by service providers who do not feel equipped to address their needs (Uink, Dodd, et al., 2023).

It is typically assumed that when accessing healthcare, Aboriginal and Torres Strait Islander LGBTQA+ people must choose between three different types of services: Aboriginal community-controlled health organizations (ACCHOs), LGBTQA+ health services, and general or mainstream health services (Day et al., 2023; Uink, Dodd, et al., 2023). Accordingly, Aboriginal and Torres Strait Islander LGBTQA+ peoples’ well-being has emerged as an area of interest for ACCHOs that seek to be more LGBTQA+ inclusive (e.g., the Aboriginal Health Council of South Australia recently became the first ACCHO to receive “Rainbow Tick” accreditation to demonstrate their capability to provide LGBTQA+ inclusive care (Aboriginal Health Council of South Australia, 2021)) and LGBTQA+ organizations that provide targeted services to Aboriginal and Torres Strait Islander people (e.g., ACON, 2023; Thorne Harbour Health, 2023). The evidence available on Aboriginal and Torres Strait Islander LGBTQA+ people’s service engagement supports the idea that they engage with both ACCHOs and LGBTQA+ health services, but also finds that they most frequently use general services, including general practitioners, psychologists, counsellors, and crisis lines (Day et al., 2022; Hill, Dodd, Uink, Bonson, & Bennett, 2022; Spurway et al., 2022). As such, there is an imperative for general services to ensure that they provide inclusive care for all communities, including explicit recognition of intersectional identities.

The research literature further points to challenges in current health service delivery recognized by both Aboriginal and Torres Strait Islander LGBTQA+ people and health service staff. Staff identify a clear need for training to increase services’ capacity to care for Aboriginal and Torres Strait Islander LGBTQA+ people (Hill, Dodd, Uink, Bonson, & Bennett, 2022). Accordingly, health consumers report that services have a generally low level of knowledge about appropriate care (Sullivan et al., 2022). Across ACCHOs, LGBTQA+ services, and general services, staff raise concerns about the unsuitability of mainstream LGBTQA+ accreditation programs for Aboriginal and Torres Strait Islander LGBTQA+ people and uncertainty about safe referral pathways (Hill, Dodd, Uink, Bonson, & Bennett, 2022; Uink, Dodd, et al., 2023). They also worry that their services lack sufficient inclusion policies or that, when these policies exist, they are not reliably upheld (Hill, Dodd, Uink, Bonson, & Bennett, 2022).

It is worth giving particular attention to the service needs of Aboriginal and Torres Strait Islander LGBTQA+ young people. Mental health difficulties in youth frequently lay the groundwork for the rest of life, and as such effective service provision during this period is crucial (McGorry & Mei, 2018; Solmi et al., 2022). The need for effective service provision during adolescence and young adulthood is amplified for LGBTQA+ young people given international trends that demonstrate that young people are “coming out” earlier than previous generations, thereby doing so during a developmental period associated with greater prejudicial attitudes and greater susceptibility to peer influence and bullying (Russell & Fish, 2016). This trend additionally means that health services are increasingly likely to encounter Aboriginal and Torres Strait Islander young people who are seeking LGBTQA+ inclusive care. Moreover, young people’s needs are often very different to their adult counterparts due to unique barriers they experience accessing care and their progressing physical, cognitive, and social development (Patton et al., 2016). For instance, preference for informal support, family beliefs about mental health, anticipated consequences such as upsetting or being removed from family, fears of being judged or not taken seriously by clinicians, and other personal and structural barriers may prevent young people from accessing mental health care (Aguirre Velasco et al., 2020; Bassilios et al., 2017; Burns & Birrell, 2014; Gulliver et al., 2010; Platell et al., 2021; Radez et al., 2021). Increased engagement of young people, and Aboriginal young people in particular, in decision-making about service provision may increase not only accessibility but also quality of care (Culbong et al., 2023; Hardy et al., 2020; Head, 2011; Wright et al., 2019). As such, effective service provision to Aboriginal and Torres Strait Islander LGBTQA+ young people has the potential to deliver substantial benefit, but requires young people’s input to ensure that organizational practices are driven by young people’s perspectives and not assumptions about their needs (Uink, Dodd, et al., 2023). To date, however, young people’s perspectives, especially those under 18 years of age, have been overlooked within Aboriginal and Torres Strait Islander LGBTQA+ research (Uink, Dodd, et al., 2023).

Of the previous research, only two studies have focused specifically on Aboriginal and Torres Strait Islander LGBTQA+ young people’s service needs, specifically social and emotional well-being (Spurway et al., 2022; Sullivan et al., 2022) and sexual health (McCormack et al., 2024) needs. These studies both took place in New South Wales. The authors found that, in addition to the issues cited in the adult literature, young people expressed a sense that services were not intended “for them” (McCormack et al., 2024). They were critical of the dichotomy between services intended for Aboriginal and Torres Strait Islander people and LGBTQA+ people, and the expectation that they would fall neatly into either service model while suppressing other parts of their identity. This made them feel invisible and distrustful of services (Spurway et al., 2022; Sullivan et al., 2022). However, young people’s engagement with sexual healthcare was potentially supported by their belonging to a shared community of Aboriginal LGBTQA+ people, which functioned as a source of resilience, strength, and knowledge (McCormack et al., 2024). In recognition of the ongoing need for research to reflect the heterogeneity of Aboriginal and Torres Strait Islander people (Walter, 2018), further research is required to confirm whether these experiences are consistent across different cultural, political, and geographical contexts.

The current study adds to the literature by contributing findings from Aboriginal LGBTQA+ young people’s perspectives on their health service needs and preferences from the context of Boorloo (Perth), Western Australia. We use the acronym LGBTQA+ in this study as it best reflects the participant demographics captured here but acknowledge the use of other terms across the research literature. This study addresses the research question: *What are the current service experiences and preferences for care among Aboriginal LGBTQA*+ *youth?* This knowledge will contribute to an evidence-based and community-led response to improve well-being outcomes for Aboriginal LGBTQA+ young people.

## Methods

### Procedure

This study forms part of the broader Walkern Katatdjin (roughly translated as “Rainbow Knowledge” in Noongar language) research project, which aims to understand the mental health and social and emotional well-being of Aboriginal and Torres Strait Islander LGBTQA+ young people through mixed-methods data collection (full research design available at https://www.rainbowknowledge.org/). This study seeks to address a stand alone research question: What are the current service experiences and preferences for care among Aboriginal and Torres Strait Islander LGBTQA+ youth? As such, only findings concerning participants’ experiences in health, mental health and social services and their identified needs for care are considered here. The project arose from community-identified need for mental health data (Liddelow-Hunt et al., 2021). As such, this study was primarily focused on service provision to support mental health and social and emotional well-being. However, this necessitates considering a large range of services given the holistic definition of social and emotional well-being used in Aboriginal health (Gee et al., 2014) and the multiple pathways through which young people may enter the mental health system (Bassilios et al., 2016, 2017; Burns & Birrell, 2014; Rickwood et al., 2014). The data reported here comes from Phase 1 of the Walkern Katatdjin project, which received ethics approval from the Western Australian Aboriginal Health Ethics Committee (WAAHEC). In line with best practice Aboriginal research, the project was overseen by a Governance Committee, consisting of respected Aboriginal LGBTQA+ community members, and a Youth Advisory Group made up of Aboriginal LGBTQA+ young people. The Governance Committee and Youth Advisory Group govern the project data according to the Walkern Katatdjin Data Governance Statement (Walkern Katatdjin, 2019), in line with the principles of Indigenous Data Sovereignty (Maiam nayri Wingara & Australian Indigenous Governance Institute, 2018). This includes thorough review of all analyses and proposed publications, decision-making concerning Indigenous Cultural and Intellectual Property, and quarterly meetings in line with the Research Collaboration Agreements between the Governance Committee, Youth Advisory Group, and research team. This study follows the CONSIDER statement criteria for reporting Indigenous peoples’ health research (Huria et al., 2019) in recognition of the need for more rigorous reporting on research with Indigenous peoples in order to reduce exploitation of Indigenous data.

Participants were eligible to participate if they were Aboriginal and/or Torres Strait Islander LGBTQA+ young people aged 14–25 years old living in the Boorloo (Perth) metropolitan area and Binjareb boodja (Peel region) of Western Australia. Participants were recruited through posts on social media, and flyers and posters displayed in mainstream, LGBTQA+, and Aboriginal medical and mental health services, universities, technical and further educations institutions (TAFEs), youth centers, and public notice boards. All participants were required to give informed consent to participate in the study by signing a written consent form witnessed by a member of the research team prior to interviews/focus groups commencing. Parent/guardian consent was not required. Participants aged 14–18 completed a screening with a clinically trained member of the research team to determine their capacity to provide informed consent to participate in research.

Data collection consisted of semi-structured interviews and yarning-style focus groups (Bessarab & Ng’andu, 2010), carried out in late 2019 and early 2020. In total, 14 Aboriginal LGBTQA+ young people participated in the study. Seven participants completed both a one-on-one interview and a focus group, six completed an interview only, and one participant took part in a focus group only. Interviews ran for 1–2 hours, and the majority were facilitated by a young Aboriginal LGBTQA+ research assistant (SLH). Two interviews were led by an Aboriginal researcher (BU) accompanied by SLH Focus groups ran for approximately 3 hours and consisted of 3–4 participants. The focus groups were facilitated by SLH and BU. Of those who participated in both an interview and focus group, most completed the interview first. As such, focus groups centered collaborative discussion of key themes the participants identified as significant, whereas interviews elicited personal narratives according to a pre-determined set of interview questions. Focus groups began with participants introducing themselves and brainstorming factors that influenced their well-being and experiences in healthcare, which the facilitators then used to guide discussion.

Interviews and focus groups were audio recorded and transcribed by a professional service. Transcripts were then sent back to participants for member checking prior to analysis; only four participants responded. No participants requested changes to their original transcript, but one participant decided to complete a second interview to discuss how their views had since changed.

The interviews and focus groups covered a broad range of topics participants saw as significant to their well-being including personal identity, family relationships, and cultural connection, which are reported in Liddelow-Hunt et al. (2023). Findings from Liddelow-Hunt et al. (2023) identify family, community, representation, and stigma as priorities for Aboriginal LGBTQA+ young people’s well-being. This study seeks to address a stand alone research question through analysis of the data concerning services experiences, predominantly focused on participant responses to three specific interview questions:

1. Can you tell us any experiences you have had accessing health services (including mental health services)?
2. Can you tell us any experiences you have had accessing social welfare services?
3. If you had a magic wand/magic stick, what would services be doing?

This component of the data is conceptually distinct and has differing implications for policy and practice, and therefore was analyzed separately. Findings addressing this research question can be directly translated into recommendations for service provision, in line with the responsibility to support Indigenous advancement (Huria et al., 2019), unlike previous findings that explore well-being concepts and experiences. Additionally, using the same interviews/focus groups to address two distinct research questions was a logistical consideration to avoid placing undue burden on a small cohort of young people. While there is a dearth of research concerning Aboriginal LGBTQA+ young people’s unique needs, Aboriginal communities as a whole are currently overburdened with requests for research participation, and good practice research must address this concern in its design (Huria et al., 2019).

### Data Analysis

The data was analyzed using reflexive thematic analysis (Braun & Clarke, 2006), adapted to an Aboriginal context by using Indigenous standpoint theory (Moreton-Robinson, 2013) as the framework for reflexivity. As such, the research team generated common themes across the dataset according to patterns of meaning they identified within the data. All transcripts were coded in NVivo by SLH, with the codebook created inductively based on the data. Four other researchers (AL, YP, BH and BU) were given data samples and the research team met to check their interpretation of the data against the codebook and collaboratively develop themes. Analysis tended more descriptive, given the dearth of existing research specific to Aboriginal LGBTQA+ young people and the imperative to safely translate young people’s perspectives into improved service provision. Nonetheless, the major themes generated represent the research team’s interpretation of meanings that structure the data. The research team consisted of a young queer Wajarri person (first author), a straight cisgender Noongar woman, a gay cisgender Nyungar (Wardandi) man, a queer cisgender non-Indigenous woman, and a straight cisgender non-Indigenous woman. Utilization of Indigenous standpoint theory acknowledges that each team members’ positionality as “situated knowers” affected their interpretation of the data. For Aboriginal researchers, this positionality further incorporated their relationships with Country, community, and kin, within which the research process is situated (Moreton-Robinson, 2013).

Findings were sent back to participants for further member checking in the form of a recorded presentation of results to ensure that they had the opportunity to question any findings that they felt did not reflect their contributions to the research. Only one participant responded to confirm they were happy with the results and did not provide further input. Findings were also reviewed by the Governance Committee and Youth Advisory Group, and then by partner organizations and other stakeholders (including Aboriginal community-controlled organizations, LGBTQA+ services, and a mainstream service), with only minor feedback. This review process helped to confirm that the findings were a credible and responsible representation of the experiences of Aboriginal LGBTQA+ young people.

## Results

### Participants

Fourteen Aboriginal LGBTQA+ young people aged between 14 and 25 years living in the Boorloo (Perth) metropolitan area and Binjareb boodja (Peel region) of Western Australia participated in the study. Table 1 (below) describes participant demographics. Although the study was open to Aboriginal and/or Torres Strait Islander people, there were no Torres Strait Islander participants. Most participants were from Western Australian cultural groups. As such, the results of the study predominantly reflect Western Australian Aboriginal experiences and perspectives, with a particular focus on those young people currently living in a major city.

**Table 1.** Participant Demographics (*N* = 14).

Demographic	*n*
Gender	
Cis woman	10
Cis man	3
Non-binary	1
Sexuality	
Gay/lesbian	6
Bisexual	4
Pansexual	4
Queer	1
Grew up in…	
Perth/South-West	9
Regional/remote Western Australia	4
Interstate	2
Cultural group	
Noongar (South-West Western Australia)	4
Other Western Australian cultural group	7
Interstate cultural group	1
Unsure or preferred not to say	2
Total	14

#### Service Use

Participants had accessed a range of Aboriginal, LGBTQA+, and mainstream health services. Discussions were mostly about primary care or mental healthcare, with some discussion of hospital emergency departments, social services, and school supports.

### Themes

Analysis identified the following themes and subthemes (Table 2):

**Table 2.** Themes (Bolded) and Subthemes.

**Unmet need for “whole self” care**
Narrow focus of LGBTQA+ services
Discomfort in ACCHOs
Preference for Aboriginal and/or LGBTQA+ health workers
Disconnection between services
Doubting services’ ability to help
**Communicating to young people that they will be welcome, safe, and cared for**
Outreach to address shame, mistrust, and confidentiality
Uncertainty about what is available and how to access it
Visual signifiers of allyship and safety
**Engaging communities to address structural inequalities**

### Unmet Need for “Whole Self” Care

I feel like if you do go somewhere for mental health support, it’s LGBTQA+ and then Indigenous people. There’s no support or information for those struggling with both. It’s like you’re one or the other.

A dominant theme concluded from participants’ stories was the need for services that would support their “whole self.” The siloing of service provision into Aboriginal or LGBTQA+ healthcare and the generally low level of knowledge about these intersecting identities among clinicians in all service types made participants feel as if they needed to compartmentalize parts of their identities and experiences when accessing care. As such, the services they received did not sufficiently address their needs. Participants described a process of negotiating which aspects of themselves they could express in any given health setting and which needed to be suppressed.

Participants felt that creating services, spaces, and resources specifically for their demographic would help them to discuss how the complexities of being Aboriginal, LGBTQA+, and young impacted on their well-being, for example, navigating kinship as a sexuality diverse person or unpacking how colonial policies have impacted on community leaders’ understandings of LGBTQA+ identity.

Probably anything to do with being able to just call someone and talk through how I’m feeling, and having them understand the Indigenous part of it … That would have made it so much easier to reconcile being Indigenous and being LGBT …

These are layered and sometimes painful issues that young people frequently have to navigate alone or with minimal support. Participants said that yarning groups specifically for Aboriginal LGBTQA+ people, peer support, chat services, or targeted resources would all be appropriate forms of support.

#### Narrow Focus of LGBTQA+ Services

LGBTQA+ health services should represent an opportunity for Aboriginal LGBTQA+ young people to receive specialized and reliable LGBTQA+ inclusive care, but participants felt that their Aboriginality was ignored when they visited LGBTQA+ services, thwarting the ability of these services to provide support for participants’ whole selves. At the LGBTQA+ services they accessed, there were no resources available specifically for Aboriginal people and no information addressing cultural concerns that they wanted support navigating.

… for one, they weren’t culturally competent. They didn’t really know anything about that kind of stuff; and two, they only really catered for lesbians and gays, and sometimes transgender if you were really lucky … So it’s hard to go to services that don’t actually cater for you on the two fronts, is that they don’t actually understand you culturally, but they also then don’t even understand you in the service that they’re trying to provide to you.

Participants also noted that the LGBTQA+ services they had accessed primarily catered for lesbian and gay people. As such, some participants felt that, in addition to not supporting their cultural identity, LGBTQA+ services provided inadequate support for their sexuality or gender identity.

#### Discomfort in ACCHOs

ACCHOs are among the limited spaces where young people can receive culturally informed healthcare, engaging cultural aspects of Aboriginal LGBTQA+ young people’s selves, but participants demonstrated a level of discomfort and dissatisfaction engaging with these services. Participants reported mixed quality of care at ACCHOs. No participants had experienced outright homophobic or transphobic discrimination while accessing an ACCHO. Some participants were concerned about the possibility of discrimination in Aboriginal spaces more generally, but this did not seem to have informed their decisions about which services to access. Several participants expressed concerns about confidentiality when accessing Aboriginal services, particularly in relation to their sexual health. These fears were specifically tied to the number of staff who were also part of the local Aboriginal community and therefore known to participants.

… they’re going back to office where everyone is and they’re blabbering and telling everyone’s personal information. And then when you go out to the community and then that person knows what’s going on with you.

More commonly, participants felt excluded from Aboriginal services because they were fair-skinned or not from the community in which the service was operating. These participants felt that accessing Aboriginal services made them feel uncomfortable or ashamed, they were afraid of being declined service, and felt that they were being judged or de-prioritized by staff at the service.

I personally, from my experience, being fair-skinned, growing up on a Country that isn’t mine, where I’m not related to every single person here, I wouldn’t feel comfortable going there ‘cause I get a lot of judgment even in my workplace. I work in a not-for-profit in Aboriginal health and there’s a lot of lateral violence …

#### Preference for Aboriginal and/or LGBTQA+ Health Workers

Seeing their intersectionality reflected among the staff at services may facilitate Aboriginal LGBTQA+ young people expressing and receiving care for their whole selves.

… it makes you feel a bit more comfortable in my opinion, and then I feel like we’ve got a connection already. I can talk about it comfortably ‘cos I feel like you might understand or at least understand more than someone who doesn’t have that kind of background …

Participants overwhelmingly agreed that they wanted the option of receiving support from a healthcare worker who was Aboriginal and LGBTQA+. They said this would make them feel comfortable, foster a sense of connection, and that someone who shared their experiences would be better able to understand them and provide support for their “whole self.” They also stated that it was helpful to see a healthcare worker who was Aboriginal *or* LGBTQA+ and that they had had positive experiences with such as counsellors, psychologists, and social workers.

I finally found an Indigenous psychologist which makes such a difference because I don’t think someone who’s not a person of colour can necessarily understand at the depth that you need.

However, participants had only been able to identify a small number of Aboriginal and/or LGBTQA+ mental health professionals, meaning that their options were limited.

#### Disconnection Between Services

Perceived competition and disconnection between services was viewed as one of the underlying causes for the lack of whole-self-care participants required. Participants perceived services as competing for funding instead of working together to provide the best possible care. They felt that better connection between services would be a step toward providing “whole self” care by making better use of existing resources and expertise, and providing a more holistic model of care.

… you could find a youth service like this, but then there’s no way for you to connect from this to another service that helps with the gender-specific issues … It could be good to have just the one health and wellbeing and housing and everything. At the moment, you have to go through three different services to understand how to get the all-around support.

They also stated that more connected services would help them to deal with complex issues rather than being required to navigate multiple services, noting that the current siloed model did not reflect the multi-faceted and intertwined nature of their well-being. The other solution participants proposed to address this issue was multiple co-located services or a “one-stop-shop,” which would similarly require increased collaboration between services with differing expertise.

#### Doubting Services’ Ability to Help

Some participants stated that, because of the generally low level of knowledge about providing care for Aboriginal LGBTQA+ people and lack of Aboriginal LGBTQA+ clinicians, they doubted that health workers would understand their experiences and, as such, would be unable to help them.

I do access a psych which I find very helpful, but I don’t know if she can fully understand my experience being a European white female. You know? So, in terms of my brain stuff, she’s probably real good, but I don’t know if she’d fully understand all the other things that come along with being part of the Aboriginal and rainbow communities.

Because of this perceived inability to provide appropriate care, several participants chose not to access mental health services.

I’ve never reached out to any of the other services just because I’m like “I don’t know if you’re gonna understand this space.”

### Communicating to Young People That They Will Be Welcome, Safe, and Cared For

So I think having something—a service that’s openly like, “We’re here for LGBTQI+ people in the Aboriginal community,” and is specifically targeting us is the best thing because like you can go down to [ACCHO], you can go to a GP, but it’s just general, like, it’s not for you, it’s for everyone. And you don’t know what you’re walking into. You don’t know if it’s gonna be safe for you …

The barriers to accessing healthcare as an Aboriginal LGBTQA+ young person were primarily around feeling unwelcome or undeserving of care, or being unaware of what services were available to them. They felt that these barriers could be reduced if services better communicated to Aboriginal LGBTQA+ young people that their service existed and would be safe for them to access.

Participants additionally reported some hesitancy accessing care due to previous experiences of racism and/or homophobia. They also experienced barriers relating to cost, location, waitlists, and the limited number of services in regional or remote areas.

I’ve been through that process of realizing you need help, to go see a therapist, waiting the whole waitlist, going to see someone, it not really working out and then going again. It’s really tiring when you’ve realized you need help and also want it but you can’t reach it. I’m also only able to access this because the therapist that I go to is a free organization …

These issues, while common among many young people (Aguirre Velasco et al., 2020; Platell et al., 2021; Radez et al., 2021), may be further exacerbated among Aboriginal LGBTQA+ young people, as they are layered on top of the additional barriers and unmet needs Aboriginal LGBTQA+ young people face accessing services.

#### Outreach to Address Shame, Mistrust, and Confidentiality

Mistrust is a barrier to Aboriginal LGBTQA+ young people’s service access that could be addressed by undertaking outreach activities to ensure they feel welcome and understand that their privacy will be respected when receiving care. Some participants stated that they did not access services or were selective with what they disclosed to clinicians because they were unsure whether their confidentiality would be respected. This included fears of being “outed” by healthcare workers to parents/caregivers and feeling unable to discuss family issues in small towns where clinicians were known to their family.

They asked me [if I was LGBTQA+]. I never told them just because I had the fear of my parents finding out and that was the main thing for me, but also friends as well, close ones that are like family, I didn’t wanna lose them.

For those who had a background in healthcare or had received good education from their clinician regarding patients’ rights, this fear was reduced. This indicates that confidentiality as a barrier to access is largely related to participants’ rights in healthcare not being communicated to them. This is a missed opportunity, given that other participants felt the confidentiality and objectivity provided by their mental health professionals contributed to what they considered an overall positive impact from receiving care. Some participants with good knowledge of confidentiality requirements still voiced concerns about these requirements not being upheld by service staff (most prominently in ACCHOs), but this does not appear to have deterred them from accessing care.

#### Uncertainty About What Is Available and How to Access It

As Aboriginal *and* LGBTQA+ *and* young, participants had many options for receiving specialist services (including but not limited to ACCHOs, LGBTQA+ health services, and youth services), which they were not previously made aware of. When asked about accessing different types of services, especially LGBTQA+ health services, participants frequently stated that they “didn’t know what that was.”

Participant 1 … the only reason I actually know about [mental health service] is because my school actually advocates them a lot which is good. But for a very long time, I didn’t even know they were like a free organization and once they were free, I was like …

Participant 2 I may google them after, tonight.

Participant 3 Yeah, same.

During the yarning groups, participants made service recommendations to each other and remarked on the list of services provided to them as part of the study’s distress protocol, noting that there was more support available to them than they had previously been aware of. Participants stated than in order to address this, services needed to do more outreach in schools, TAFEs and universities, where they would best be able to reach young people.

#### Visual Signifiers of Allyship and Safety

Visual signifiers were a much-discussed avenue for removing the guesswork involved in determining whether a service would be welcoming, competent, and safe for Aboriginal LGBTQA+ young people. This uncertainty about whether services were culturally safe and/or LGBTQA+ inclusive made participants anxious and could deter them from seeking help or disclosing either their Aboriginality or their sexuality or gender to health workers. In order to remove that guesswork, participants said that services needed to be vocal about their support for Aboriginal and LGBTQA+ people. This included through displaying flags, posters, and pamphlets portraying Aboriginal and LGBTQA+ people.

But having that representation and being clear with what the service is offering, taking away that mystery and making it a normal safe space like this is …

Participants also frequently used intake forms as a way of gauging how LGBTQA+ inclusive a service was. Intake forms with options for indicating a non-binary gender or pronouns indicated that a service would be safe and competent. Participants also thought that visibly displaying signs of LGBTQA+ inclusion would make homophobic and transphobic people less inclined to use the service, contributing to a broader community culture of acceptance.

I think I just—when I was doing appointments, after I made an appointment then I was just looking through their info or something, and some them will have a rainbow flag at the end of their websites and stuff like that. The GP would be really kind when it came to me talking about my gender and all that stuff.

However, participants noted that these signifiers, designed to engage marginalized communities in the service, were often superficial. What was additionally needed was healthcare providers with insight into the experiences of Aboriginal LGBTQA+ young people who were capable of providing genuine and effective support.

### Engaging Communities to Address Structural Inequalities

But it comes back to what I think I really strive for and what I have to struggle with for a lot of these services is do they have that white knight complex? Are they like, “Oh, this poor Indigenous mob, I’ve got to save them,” and put us in social deficit with them, or are they like, “I wanna help empower Indigenous mob. What do I need to do to empower you to be able to go out and do your stuff?”

Throughout the interviews and focus groups, participants reflected that their experiences were symptomatic of the broader marginalization of Aboriginal LGBTQA+ people, which impacted on services’ ability to provide trustworthy and inclusive care. Participants expressed that provision of culturally safe care went beyond engaging respectfully with individuals and required empowering communities. They broadly criticized services that did not consult the communities they supposedly serve in their decision-making.

That’s the thing, is having to hear what people think and hear what the actual people that experience the services think, instead of just having someone sitting at a desk thinking that this is the best for them and it’s like, no, you’ve got to come into the community and ask.

They further noted that paternalistic healthcare provision was a facet of colonialism and critiqued organizations designed to help Aboriginal communities that were run by non-Indigenous people. As such, they discussed how many Aboriginal communities remain distrustful of services, who need to actively work within this context to earn credibility. Building this credibility was necessary for services to be able to engage homophobic communities and safely provide LGBTQA+ inclusive healthcare.

… if you have an LGBT mob come out there to give a talk or whatever, they just have a disengaged audience and be booed off the stage because the situation is generally very homophobic. So it’s how do you safely engage people and help safely get people who understand and know about any of that sort of stuff there?

All of these discussions represented a sense among participants that the various supports available to Aboriginal LGBTQA+ people perpetuate disempowerment, are not accountable to their communities, and do not value their voices.

## Discussion

The aim of this study was to understand the current service experiences and preferences for care among Aboriginal LGBTQA+ youth. The findings shed light on the almost complete lack of Aboriginal LGBTQA+ youth–focused care available, resulting in inadequate support that may exacerbate health inequities already experienced by Aboriginal LGBTQA+ young people due to their location at the intersection of two marginalized communities. The findings prompt reflection from researchers and healthcare providers on the often paternalistic and individualistic way in which research and services still seek to engage young people in conversations about their healthcare, in direct contrast to the benefits that come from meaningful involvement of young people in decisions about health service provision and policy (Patton et al., 2016). The findings demonstrate that Aboriginal LGBTQA+ young people are highly interested in discussing their healthcare needs, how services operate and are funded, and how this occurs in the context of systemic inequality.

Participants recognized their experiences as existing within the context of settler heteropatriarchy (Day, 2021; O’Sullivan, 2021), which shapes the healthcare system to be hostile toward Aboriginal LGBTQA+ young people. This may be, for example, through overt instances of racism, homophobia, and transphobia, and more covertly through the devaluation of Aboriginal understandings of well-being and assumptions that all users are heterosexual and cisgender. The specific needs of Aboriginal LGBTQA+ people described by participants (e.g., including pronouns on service intake forms or displaying an Aboriginal flag), which may be viewed as “additional” components of care (and consequently de-prioritized), are fundamentally attempts to overcome this systemic hostility. Many of the needs articulated by participants are common among young people (Aguirre Velasco et al., 2020). For example, confidentiality, mistrust of mental health providers, and the belief that mental healthcare will not help them are frequently found to be barriers to young people’s engagement with health services (Boyd et al., 2011; Charman et al., 2010; De Anstiss & Ziaian, 2010; Rughani, 2011; Yap et al., 2011). However, these barriers are exacerbated among Aboriginal LGBTQA+ young people because they are intertwined with the inequities created by settler heteropatriarchy’s influence on healthcare. For example, better outreach and advertising from services is additionally important because of the potential for discrimination in all types of services, which makes it necessary for Aboriginal LGBTQA+ young people to know that there are varied options available to them so that they are not forced into accessing a harmful service, or alternatively resort to avoiding healthcare. This does require additional work from services to communicate levels of safety and inclusion for Aboriginal LGBTQA+ young people, but this must be recognized as a consequence of Aboriginal LGBTQA+ young people’s marginalized placement within the Australian settler-colony.

These findings reflect many similarities with the existing literature, most prominently the unmet need for resources and health promotion specifically for Aboriginal LGBTQA+ people (McCormack et al., 2024; Spurway et al., 2022; Sullivan et al., 2022; Uink et al., 2020). They also add further evidence that visual signifiers of support can help to indicate to Aboriginal and Torres Strait Islander LGBTQA+ people that they will be welcome at a service (Hill, Dodd, Uink, Bonson, Bennett, et al., 2022; McCormack et al., 2024; Spurway et al., 2022; Uink, Dodd, et al., 2023) which, along with accessibility and word-of-mouth, inform young people’s decision-making around service use and re-use (McCormack et al., 2024; Sullivan et al., 2022). Significantly, these results dispute the assumption that Aboriginal LGBTQA+ young people choose not to engage with ACCHOs because of fear of anti-LGBTQA+ discrimination (Dunn/Holland et al., 1994; Hope & Haire, 2019). While participants did acknowledge potential homophobia and transphobia in ACCHOs, their decision-making was more informed by accessibility and feeling unwelcome because they lacked local community connections and fear of lateral violence. Further, compared to previous research, findings from this study support the need for a shift away from white normativity in LGBTQA+ services (Sullivan et al., 2022) *and* point to a need to recognize the diversity of sexuality and gender identities among Aboriginal LGBTQA+ young people. This should be supported by further research and health promotion focused on Aboriginal pansexual, asexual, and non-binary people.

Most significantly, the results point to the importance of health workers and, especially, mental health professionals understanding the broader sociohistorical context that impacts young people’s well-being. This aligns with the social and emotional well-being approach (Gee et al., 2014) frequently adopted in Aboriginal research and health practice, which centers connections that are shaped by social, cultural, political, and historical contexts. In addition to awareness of the complex impacts of colonization on community attitudes toward sexuality and gender diversity, which was noted by participants as a key conversation for health workers to be capable of engaging with, it is also important to understand the current issues that impact Aboriginal LGBTQA+ young people and how these will differ between individuals. These contexts must be recognized as the foundation of Aboriginal LGBTQA+ young people’s health. This need for a deeper understanding was reflected in participants’ preference to receive care from another Aboriginal and/or LGBTQA+ person, who hypothetically would already possess this knowledge. Lived experience staff also forms part of imagined healthcare for Aboriginal LGBTQA+ young people in sexual health (McCormack et al., 2024), and there is evidence to support the efficacy of peer-led support and health promotion for young people (Savaglio et al., 2023) and Aboriginal young people specifically (Vujcich et al., 2018). In the absence of lived experience staff members, there is a clear and recognized need for training that is grounded in historical and local context (Wilson et al., 2023). Development of a deeper understanding of Aboriginal LGBTQA+ young people’s unique experiences and the context in which they occur stands out as a necessary skill for health workers *above* skills that are focused on by health workers themselves (Hill, Dodd, Uink, Bonson, Bennett, et al., 2022) such as using correct terminology. Participants typically discussed inclusive language as an *indicator* of inclusive attitudes and competency, rather than sufficient in itself. This additionally points to why many mainstream training and accreditation programs are inappropriate for Aboriginal people (Hill, Dodd, Uink, Bonson, Bennett, et al., 2022): because they do not equip workers to understand the broader context that informs Aboriginal people’s well-being or to engage with complicated dialogues around community attitudes toward LGBTQA+ identities.

Based on the findings of this study, we make the following recommendations for health services (table 3):

**Table 3.** Recommendations.

“Whole self” care: *Workforce*	Attract and retain Aboriginal and/or LGBTQA+ staff.
“Whole self” care: *Training*	Provide staff with training on how to work with Aboriginal LGBTQA+ young people. This should include
	• Recognizing the diversity among Aboriginal LGBTQA+ young people
	• Understanding the sociohistorical and current contexts that impact Aboriginal LGBTQA+ young people’s well-being
Communicating to young people that they will be welcome, safe, and cared for: *Signs*	Use visual signifiers of support (e.g., flags and posters) to indicate that the service is inclusive.
Communicating to young people that they will be welcome, safe, and cared for: *Resources*	Create or share specific resources and health promotion for Aboriginal LGBTQA+ young people. This could include
	• Up-to-date information about LGBTQA+ identities and health
	• Clear information about confidentiality and the circumstances in which confidential information can be shared
Engaging communities: *Decision-making*	Engage Aboriginal LGBTQA+ young people in decisions about how healthcare services operate.

## Limitations

These findings should be considered within the context of the strengths and limitations of this study. The sample size of 14, while suitable for the research question given the richness of the data, is relatively small. There was an underrepresentation of men (*n* = 3) and trans people (*n* = 1); as such, these results primarily reflect the perspectives of cisgender lesbian, bisexual, and pansexual women. Additionally, most participants described themselves as moderately well at the time of participating in this study, which may reflect recruitment bias. Those young people experiencing current significant hardship or distress may not have been interested in being involved in research, and as such their experiences are absent from these findings.

As a qualitative study, the results are not generalizable beyond the cultural and geographic context of Boorloo. While our ability to comment on the experiences of youth outside the region, as well as on the experiences of Torres Strait Islander LGBTQA+ youth, is limited, the geographical specificity aligns with arguments that services must adapt their inclusive practice to meet the expectations of the local community (Uink, Liddelow-Hunt, et al., 2023; Wilson et al., 2023).

## Conclusion

In conclusion, this study adds to the literature demonstrating that, while many Aboriginal LGBTQA+ young people have positive experiences of receiving care for their health and well-being, there persists a feeling of being unable to wholly exist in healthcare settings. To address this, services must actively welcome Aboriginal and Torres Strait Islander LGBTQA+ young people and be willing to engage with the multiple intersecting contexts that shape young people’s well-being and the broader structures within which healthcare as an institution is situated.
